# Flexible Transparent Electrode Based on Ag Nanowires: Ag Nanoparticles Co-Doped System for Organic Light-Emitting Diodes

**DOI:** 10.3390/ma17020505

**Published:** 2024-01-20

**Authors:** Ziye Wu, Xiaolin Xing, Yingying Sun, Yunlong Liu, Yongqiang Wang, Shuhong Li, Wenjun Wang

**Affiliations:** 1 School of Physical Science and Information Technology, Liaocheng University, Liaocheng 252059, China; qw2320329838@163.com (Z.W.); 15684193393@163.com (X.X.); 17353609715@163.com (Y.S.); liuyunlong@lcu.edu.cn (Y.L.); wyq2385993945@163.com (Y.W.); 2 Shandong Provincial Key Laboratory of Optical Communication Science and Technology, Liaocheng 252059, China

**Keywords:** flexible organic light-emitting diode, flexible transparent electrode, argon plasma treatment, Ag NWs: Ag NPs co-doped system

## Abstract

Flexible organic light-emitting diodes (FOLEDs) have promising potential for future wearable applications because of their exceptional mechanical flexibility. Silver nanowire (Ag NW) networks are the most promising candidates to replace indium tin oxide (ITO), which is limited by its poor bendability. In this study, three different methods including methanol impregnation, argon plasma treatment, and ultraviolet radiation were used to reduce the junction resistance of Ag NWs to optimize the flexible transparent electrodes (FTEs); which were prepared using Ag NWs and poly(3,4-ethylenedioxythiophene): poly(styrenesulfonate) (PEDOT: PSS). Then, the optoelectronic properties of the FTEs were further improved by using a co-doped system of silver nanowires and silver nanoparticles (Ag NPs), the structure of which consisted of PET/Ag NWs: Ag NPs/PEDOT: PSS/DMSO. The largest FOM value of 1.42 × 10^−2^ ohm^−1^ and a low sheet resistance value of 13.86 ohm/sq were obtained using the optimized FTEs. The prepared FOLED based on the optimized FTEs had a luminous efficiency of 6.04 cd/A and a maximum EQE of 1.92%, and exhibited no observed decline in efficiency when reaching maximum luminance. After 500 bending tests, the luminance still reached 82% of the original value. It is demonstrated that the FTEs prepared via the co-doped system have excellent optoelectronic properties as well as high mechanical stability.

## 1. Introduction

Organic light-emitting diodes (OLEDs) have progressed rapidly since the first simple device was prepared on a rigid glass substrate by Tang et al. in 1987 [1]. Nowadays, with the rapid development of science and technology, flexible light-emitting diodes (FOLEDs) have gained much attention to meet the public’s demand for flexible displays and lighting [2]. However, indium tin oxide (ITO), as the most widely used electrode material, has many disadvantages in the application of FOLED devices, such as a lack of natural resources, a complicated and time-consuming production process, a high price, and an undesirable flexibility due to its inherent brittleness [3]. Many alternatives to ITO have been developed for the preparation of FOLEDs, such as carbon nanotubes [4], graphene [5], metal nanowires [6], and conductive polymers [7].

Among these alternatives, carbon nanotubes (CNTs) are suitable for industrial roll-to-roll processes, which can reduce the production cost of flexible transparent electrodes. However, the large contact resistance of carbon nanotubes limits the conductivity of the flexible transparent electrodes (FTEs) prepared with them [8,9]. Graphene is a material with high light transmittance, high electrical conductivity, and excellent flexibility, but its high cost makes it difficult to achieve the large-scale industrial production of graphene FTEs [10,11]. Conductive polymers, especially poly(3,4-ethylenedioxythiophene): poly (styrenesulfonate) (PEDOT: PSS), have high transmittance, high mechanical flexibility, and excellent thermal stability in the visible light range [12,13]. Metal nanowires, especially silver nanowires (Ag NWs), are inexpensive to synthesize and have high conductivity, so they are considered some of the most ideal materials for processing FTEs [14,15]. However, Ag NW networks face the dual challenges of having high junction resistance and high surface roughness. As a result, several post-treatments have been put forward to reduce the high junction resistance, such as thermal treatment [16], mechanical pressing [17], capillary force [18], chemical deposition [19], light-induced welding [20], etc. In order to avoid the harmful effects of rough surfaces, the roughness optimization of Ag NWs is usually carried out using conductive material coverage [21] or substrate transfer [22].

Although the above treatments can effectively improve optoelectronic properties of FTE, the efficiency of their prepared FOLEDs is still unsatisfactory. Silver nanoparticles (Ag NPs) have local surface plasmon resonance (LSPR) and a high scattering efficiency in visible light, which can improve the performance of flexible devices [23,24]. Therefore, it is meaningful to develop an FTE containing a Ag NWs: Ag NPs structure to improve the performance of FOLEDs.

In this study, three different methods were used to solve the problem of the high junction resistance of Ag NWs: methanol impregnation, argon plasma, and ultraviolet radiation. Ag NWs film was coated with DMSO-treated conductive polymer PEDOT: PSS to reduce the roughness. By comparing the performance of the FTE, which was optimized using three Ag NW treatment methods, with argon plasma treatment as the foundation, the Ag NWs: Ag NPs co-doped system was designed to further enhance the performance of FOLEDs.

## 2. Experimental Section

### 2.1. Materials

The aqueous solution of PEDOT: PSS (Clevios^TM^PH1000 and Clevios P VP.AI 4083) used in the experiments was purchased from Xi’an Bao Lite Optoelectronics Technology Co., Ltd., (Xi’an, China), which was dispersed in water at a concentration of 1.0–1.3 wt%. Ag NWs (dispersed in ethanol solution at a concentration of 10 mg/mL) were purchased from Nano New Material Technology Co., Ltd., (Guangzhou, China), which had an average diameter of 50 nm and a length of 50 μm. Super-dry DMSO solvent was purchased from J&K Scientific (Beijing, China), which had purity > 99% and water ≤ 30 ppm. A super-dry methanol solvent was purchased from J&K Scientific (purity 99.9%, water ≤ 30 ppm). N,N′-Bis-(1-naphthalenyl)-N, N′-bis-phenyl-(1,1’-biphenyl)-4,4’-diamine (NPB), aluminum Tris(8-hydroxyquinolinate) (Alq_3_), 4,7-diphenyl-1,10-phenanthroline (Bphen), and lithium fluoride (LiF) were purchased from Xi’an Bao Lite Optoelectronics Technology Co., Ltd. Al was purchased from Fuzhou Innovation Photoelectric Technology Co., Ltd., (Fuzhou, China) ITO glass (sheet resistance ≤ 15 ohm/sq; transmittance ≥ 86%) and PET substrates were purchased from South China Science & Technology Company Ltd., (Shenzhen, China).

**Figure 1:**
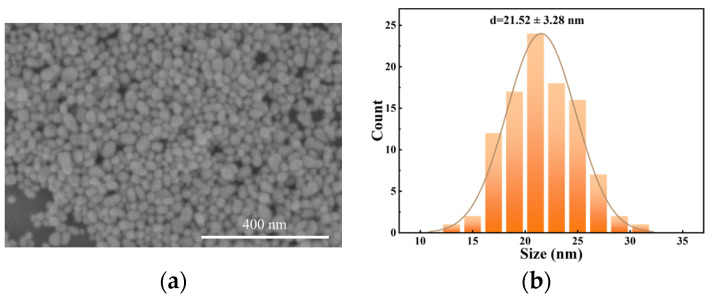
(**a**) The SEM image and (**b**) the size distribution statistics of the synthesized Ag NPs.

The Ag NPs were prepared following the specific steps outlined below. We added 5 mL of 10 mM silver nitrate (AgNO_3_) and 15 mL of 10 mM sodium citrate (Na_3_C_6_H_5_O_7_) to 90 mL of ultrapure water. The mixture was then vigorously stirred for 10 min. After the drop-wise addition of 5 mL of 10 mM sodium borohydride (NaBH_4_), the color of the solution changed to yellow, and it was stirred for 2 min. Finally, the nanoparticles were purified by centrifugation. Figure 1a shows the SEM image of the Ag NPs prepared on a silicon substrate. The Ag NPs were spherical with an average particle size of about 21 nm.

### 2.2. Characterization

To investigate the performance of the prepared FTE, the sheet resistance was tested using a 4-Point Probes Resistivity Measurement System (RTS-5, 4 Probes Tech, Fort Worth, TX, USA). The transmittance of the electrodes was tested using a UV–Vis spectrophotometer (U-3310), and the surface morphology of the films was tested using atomic force microscopy (AFM, Bruker Nanoscope V atomic force microscope, Rosemont, IL, USA) and scanning electron microscopy (SEM, ThermoFisher Apreo S HiVac, Brno, Czech Republic). The optoelectronic properties of the FOLED devices were measured using an OLED test system, the Keithley 2400, and a PR655. The samples were measured under atmospheric conditions, without any encapsulation of the device.

### 2.3. Preparation of FTEs

**Figure 2:**
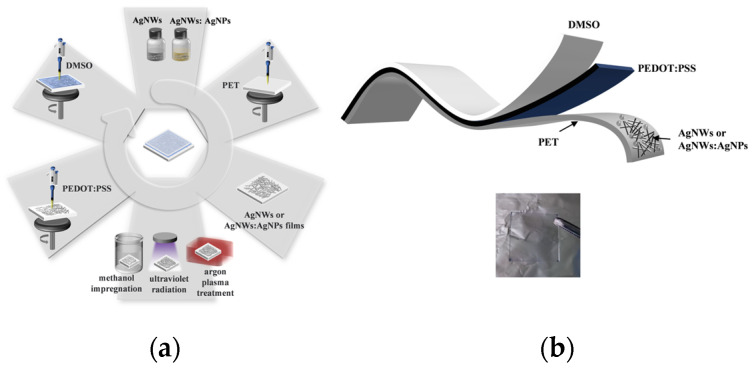
(**a**) The schematic of FTE preparation: PET substrate spin-coating Ag NWs: Ag NPs solution; Ag NWs: Ag NPs films with methanol impregnation, argon plasma, or ultraviolet radiation treatment; the Ag NWs: Ag NPs films spin-coated with PEDOT: PSS and DMSO, respectively. (**b**) Schematic diagram of FTE structure (insert, the real product picture).

In the experiments, polyethylene terephthalate (PET) was used as a flexible substrate. PET substrates (size 2 cm × 2 cm) were ultrasonically cleaned with ultrapure water and ethanol for 20 min, dried under nitrogen, and then treated with oxygen plasma for 10 min to improve the hydrophilicity of the substrates. The Ag NW dispersion (10 mg/mL) was diluted to 3.5 mg/mL with anhydrous ethanol. The diluted Ag NW dispersion and the synthesized Ag NPs were doped in a 2:1 ratio and set aside. The preparation of the Ag NW film was accomplished through a two-step spin-coating process, where the rotation speed was set to 1000 rpm for 40 s. (The Ag NWs: Ag NPs film was spin-coated at 3000 rpm for 30 s and then annealed at 90 °C for 20 min.) Subsequently, the Ag NW film was annealed at a temperature of 90 °C for 20 min. The prepared Ag NW film or the Ag NWs: Ag NPs film was optimized via methanol impregnation, argon plasma (29.6 W), or treatment with ultraviolet radiation (16 W/365 nm). Subsequently, the film was spin-coated with PEDOT: PSS solution at 3000 rpm for 40 s and then annealed at 90 °C for 20 min. Then, DMSO solution was spin-coated at the same speed. Figure 2a depicts a schematic of the FTE preparation; Figure 2b depicts a schematic diagram of the FTE structure.

### 2.4. Preparation of FOLEDs

The prepared FTEs were patterned using anhydrous ethanol. PEDOT: PSS (Clevios P VP.AI 4083) (50 nm) as a hole injection layer, was spin-coated onto the FTE and ITO surfaces, respectively, at 3000 rpm for 30 s. NPB (70 nm), Alq_3_ (60 nm), Bphen (10 nm), and LiF (0.8 nm) were thermally deposited sequentially at a controlled rate of 0.25 ± 0.05 Å/s, and Al (75 nm) electrodes were thermally deposited at a controlled rate of 1.50 ± 0.50 Å/s. The vacuum in the chamber was maintained above 5 × 10^−4^ Pa. Among them, NPB was used as the hole transport layer (HTL), Alq_3_ was used as the emitting layer (EML), and Bphen was used as the electron transport layer (ETL).

## 3. Results and Discussion

### 3.1. Characterization of FTEs

**Figure 3:**
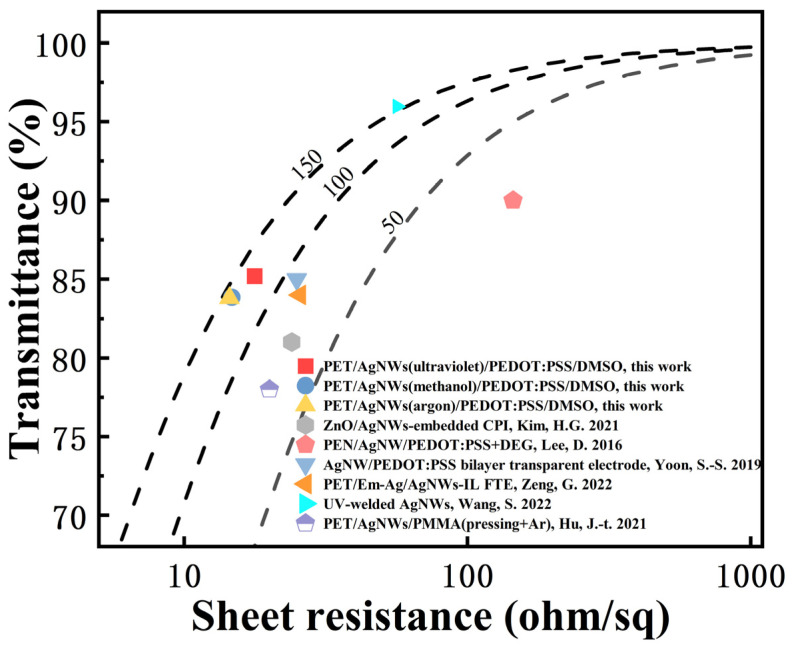
The transmittance (@ 550 nm) versus sheet resistance for the fabricated FTEs [21,26,27,28,29,30].

Three different methods, including methanol impregnation, argon plasma, and ultraviolet radiation, were used to optimize the Ag NW films to prepare various Ag NWs/PEDOT: PSS/DMSO FTEs. The sheet resistance (Rs) and the transmittance (T) of the FTEs were experimentally tested, and the results are presented in Table 1. In order to better compare the optoelectronic performance of each electrode, the Tinkham formula was introduced in this study to further characterize the relationship between T and Rs, as shown in Figure 3, and the formula is [25]:

$$T\left(\lambda \right)={\left(1+\frac{188.5}{R_{S}}\cdot \frac{\sigma_{OP}\left(\lambda \right)}{\sigma_{DC}}\right)}^{-2}$$

where $\sigma_{OP}\left(\lambda \right)$ represents the optical conductivity at 550 nm, while $\sigma_{DC}$ denotes the DC conductivity of the film. The ratio $\sigma_{DC}/\sigma_{OP}\left(\lambda \right)$ is another indicator of merit for quantitative comparisons among different electrode types. Curve fitting using the Tinkham formula was applied to $\sigma_{DC}/\sigma_{OP}\left(\lambda \right)$ values of 150, 100, and 50 (from left to right in Figure 3) and is depicted with dashed lines. The $\sigma_{DC}/\sigma_{OP}\left(\lambda \right)$ value of the FTE prepared in this study was basically around the 150 curve, which indicates advantages of both a high $\sigma_{DC}/\sigma_{OP}\left(\lambda \right)$ value and a low sheet resistance in comparison to other types of electrodes [21,26,27,28,29,30].

**Figure 4:**
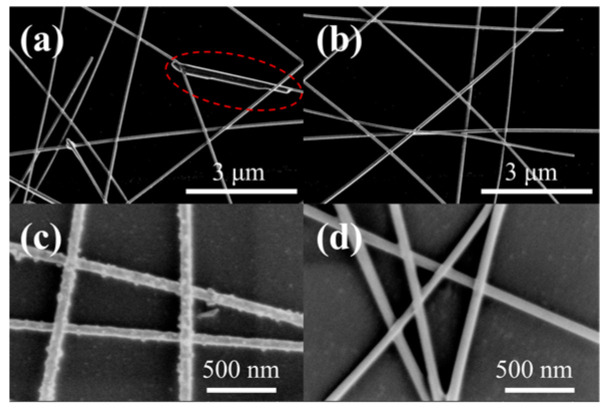
The SEM images of the Ag NW films under various treatment methods, including (**a**) untreated (PVP on the surface of silver nanowires in the red circle), (**b**) methanol impregnation, (**c**) argon plasma treatment, and (**d**) ultraviolet radiation.

**Figure 5:**
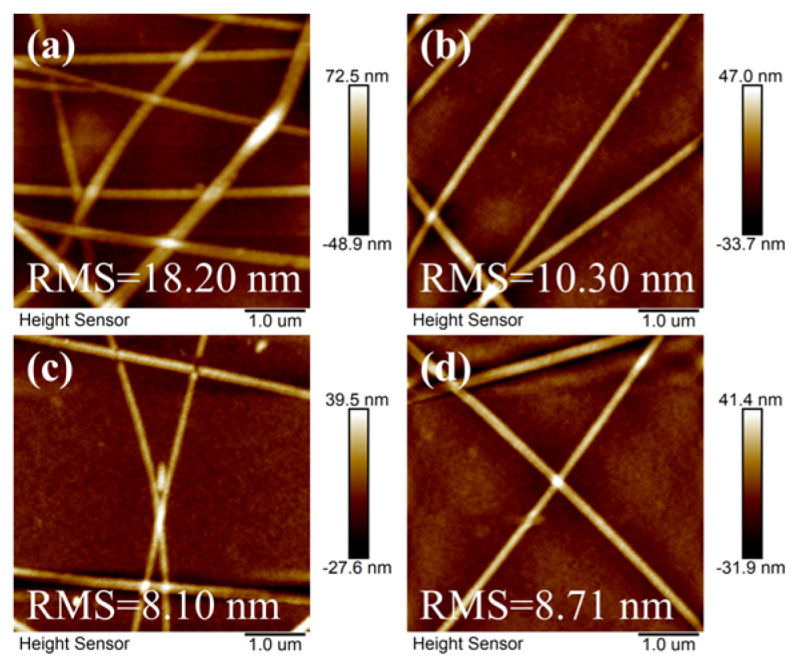
The AFM diagrams of the prepared FTEs under four different experimental conditions, namely: (**a**) untreated, (**b**) methanol impregnation, (**c**) argon plasma treatment, and (**d**) ultraviolet radiation treatment.

The scanning electron microscopy (SEM) images before and after the Ag NW films were treated in three ways are shown in Figure 4. It can be observed that with methanol impregnation treatment, most of the PVP wrapped on the surface was removed and led to a smooth surface of the Ag NWs, as shown in Figure 4b. Figure 4c,d illustrate that the Ag NWs were no longer simply stacked together after the argon plasma or the ultraviolet radiation treatment; instead, they achieved tight welding together, resulting in better contact between the nanowires. As a result, the junction resistance of the Ag NW films was reduced effectively. The surface roughness of FTE is also an important parameter for evaluating electrode performance, which has an important impact on the subsequent preparation of flexible optoelectronic devices. Therefore, AFM was used to measure the prepared FTEs, as shown in Figure 5. The root mean square (RMS) roughness decreased from 18.20 nm before treatment to 10.30 nm for the methanol impregnation treatment; to 8.10 nm for the argon plasma treatment; and 8.71 nm for the ultraviolet radiation treatment. To reduce the RMS further, a conductive polymer PEDOT: PSS was coated on the surface. Immediately, a thin layer of the DMSO solution was spun on the surface of the PEDOT: PSS to separate the PEDOT and the PSS through a charge screening phase. This dissolved the PSS at the same time, thereby releasing the conductive PEDOT for self-aggregation and improving electrical conductivity [31,32].

**Figure 6:**
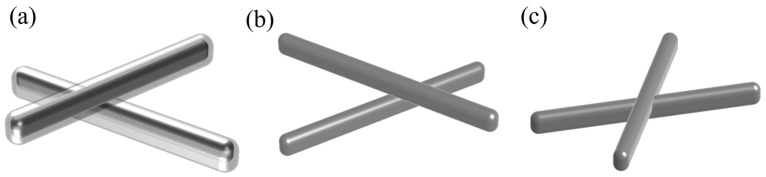
The mechanism diagram of the Ag NWs film under three different treatment methods: (**a**) untreated, (**b**) after PVP removal, and (**c**) after welding.

The phenomenon appearing in the SEM images of the silver nanowire in Figure 4, is illustrated in Figure 6. For methanol impregnation, the low boiling point of the methanol solvent made it prone to evaporation. The vapor droplets that form on the surface of the Ag NWs dissolve the surface coating PVP. The strong interaction between the methanol and the PVP molecules led to the detachment of PVP from the Ag NWs, as shown in Figure 4b and Figure 6b. For argon plasma treatment, based on the SEM images, numerous small particles were observed in the processed Ag NWs. These small particles are caused by the thermal effects of the plasma, which are a result of the impact of high-energy electrons and the heat that accumulates on the surface of the Ag NWs. As the processing time increases, the internal energy and surface temperature of the Ag NWs continue to rise, leading to their melting. After the processing is complete, the melted portion of the Ag NWs may be able to recrystallize. If the melting occurs at the intersections or adjacent areas of the Ag NWs, they can resolidify tighter during the recrystallization process [33], as depicted in Figure 6c and Figure 4c. Under prolonged illumination of ultraviolet radiation, the Ag NW networks exhibit unique and intricate behavior. The interaction of ultraviolet light with Ag NWs generates surface plasmon resonance, inducing a photothermal effect that increases the local temperature of the Ag NW network, resulting in the formation of “hot spots”. The ultraviolet light initially triggers silver ion migration and reduces the junction resistance between Ag NWs. Simultaneously, excessive light also accelerates the transformation of Ag NWs into spherical nanoparticles [34], as depicted in Figure 4d and Figure 6c.

**Figure 7:**
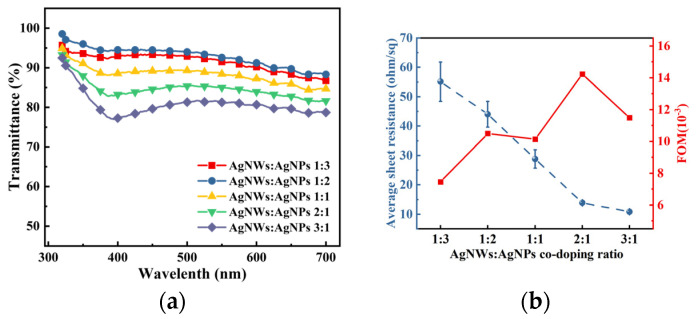
(**a**) The transmittance and (**b**) the average sheet resistance and FOM values of Ag NWs: Ag NPs for each co-doping ratio.

**Figure 8:**
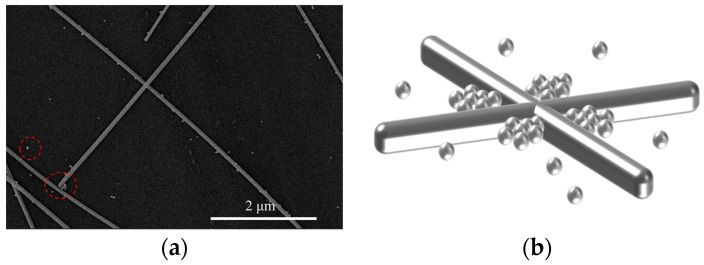
(**a**) The SEM image (randomly dispersed Ag NPs marked in the little red circle and the clusters of NWs-NPs marked in the big red circle), (**b**) the mechanistic diagram of a random dispersion of the Ag NWs: Ag NPs co-doped system.

**Figure 9:**
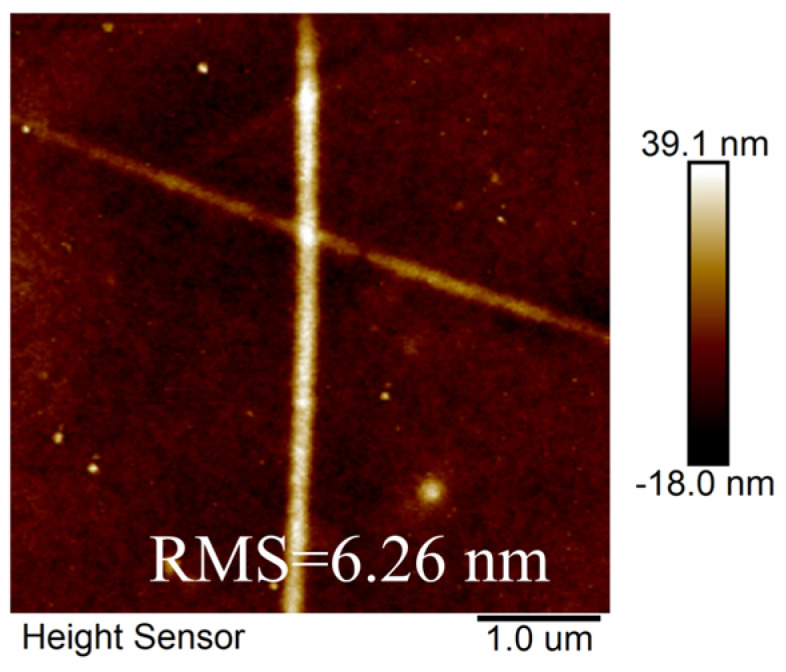
The AFM image of FTE prepared via Ag NWs: Ag NPs co-doping system.

The performance comparison of the above FTE indicates that the FTE treated with argon plasma exhibited superior performance. Building on this finding, different ratios of the Ag NWs: Ag NPs co-doped system was implemented to further enhance the photoelectric performance of the FTEs. To comprehensively assess the optoelectronic properties of the electrodes, it is important to establish a clear relationship between their electrical and optical properties. This can be achieved through the use of a figure of merit (FOM) value, which serves as a useful metric for evaluating the electrode’s overall performance. When the transmission is about 90%, the FOM value is defined as [35]:

$$\emptyset_{TC}=\frac{T^{10}}{R_{S}}$$

where $\emptyset_{TC}$ refers to the FOM, T denotes the transmittance of the electrode at a wavelength of 550 nm, and $R_{S}$ stands for the sheet resistance of the electrode. This formula is usually utilized to assess the performance of FTEs of the same type. The higher the FOM value, the better the electrode. Figure 7 presents the transmittance, the sheet resistance, and the FOM value of the FTEs at various doping ratios of Ag NWs: Ag NPs. As the concentration of nanoparticles increases, the concentration of nanowires decreases correspondingly, leading to an increase in the transmission rate of the FTEs. However, the transmittance begins to exhibit a declining trend. This can be attributed to the relatively larger gaps between the silver nanowires due to their lower content. As shown in Figure 7b, the largest FOM value of 1.42 × 10^−2^ ohm^−1^ was obtained from the Ag NWs: Ag NPs, doped at a ratio of 2:1; the average sheet resistance of the FTEs was 13.86 ohm/sq. The surface morphology of the Ag NWs: Ag NPs co-doped system was measured; the SEM image is shown in Figure 8, and the AFM image is shown in Figure 9. Figure 8a shows that some Ag NPs were randomly filled in the blank regions of the nanowire network (marked in the little red circle), and some were coupled with nanowires to form clusters of Ag NWs-Ag NPs (marked in the big red circle). An illustrative mechanism diagram was constructed, as depicted in Figure 8b. The Ag NPs filled in the interconnected gaps between the nanowires, and the doping of Ag NPs improved the electrical contacts between the nanowires [36]. Figure 9 shows that the Ag NWs: Ag NPs co-doped system had a significantly lower RMS roughness of 6.26 nm than the undoped FTEs. Good surface roughness is essential for contacting the organic layer, which is of great significance for the subsequent preparation of FOLEDs.

### 3.2. Characterization of FOLEDs

**Figure 10:**
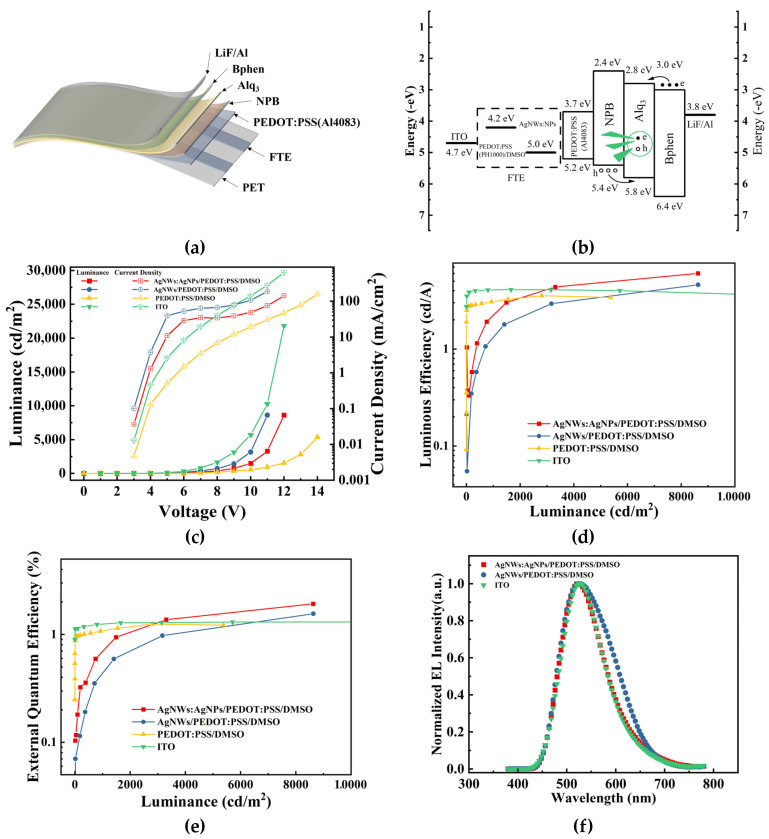
(**a**) The structure diagram, (**b**) the energy level diagram of each part (The hole, the hollow circle, and the electron, the solid circle, are combined to form excitons at the Alq_3_ and emit radiation transition luminescence), (**c**) the luminance-voltage–current density diagram, (**d**) the luminous efficiency-luminance diagram, (**e**) the external quantum efficiency (EQE)–luminance diagram, and (**f**) the normalized EL spectra diagram of the prepared FOLED devices.

With the optimized FTEs, the FOLEDs were prepared with the structure shown in Figure 10a. The energy level diagram of each part of the FOLED is shown in Figure 10b. For better comparison, the OLEDs with a rigid ITO electrode were prepared as reference devices. The FOLED device characteristics and normalized electroluminescence spectra for different electrodes are shown in Figure 10c–f. The specific performance indices are shown in Table 2. As compared with the reference OLEDs, the FOLEDs had a similar turn-on voltage, a lower luminance intensity, and a higher maximum EQE and luminous efficiency. The highest luminous efficiency of 6.04 cd/A and an EQE value of 1.92% were obtained using the FOLEDs based on the Ag NWs: Ag NPs/PEDOT: PSS/DMSO anode; both surpassing those obtained with the rigid ITO electrode of 4.10 cd/A and 1.31%, as well as the Ag NWs/PEDOT: PSS/DMSO flexible electrode of 4.60 cd/A and 1.56%. As shown in Figure 10f, the normalized EL spectra of the devices have the same peak wavelength, which means that the prepared flexible electrode has no effect on the recombination position of the OLED.

The enhanced performance of the FOLEDs based on the Ag NWs: Ag NPs/PEDOT: PSS/DMSO anode can be attributed to the inclusion of Ag NPs, which not only smooths the surface morphology of the FTEs but also improves the efficiency of light capture or extraction due to the localized surface plasmon resonance (LSPR) and the high scattering efficiency within visible light [37]. The optimized surface morphology improves the uniformity and charge transfer characteristics of the FTEs. In the FTEs with a co-doping system, Ag NWs provide transmission paths, while Ag NPs increase the specific surface area and connectivity of the conductive channels. In other words, the Ag NWs are further “welded” when Ag NPs are introduced via argon plasma treatment, thereby improving the performance of the flexible devices. Compared with the reference device, the fabricated FOLEDs with both of the FTEs did not show an efficiency roll-off when reaching maximum luminance.

**Figure 11:**
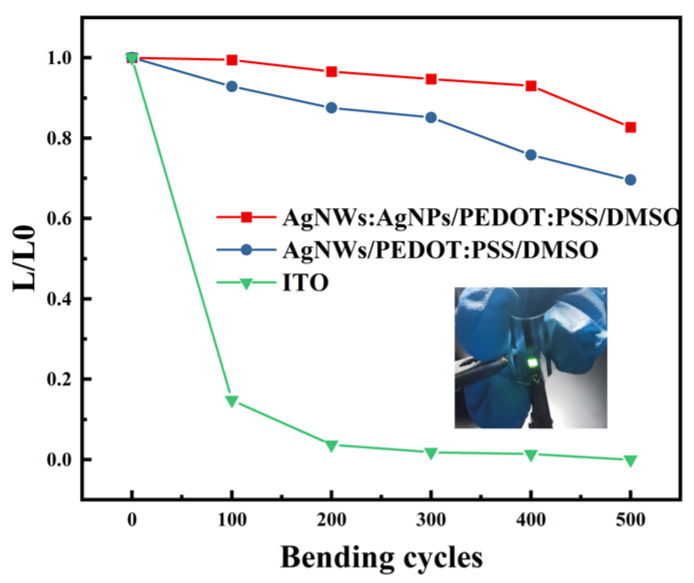
The bending test diagram of the prepared FOLED device (insert, the real product picture); L (device luminance after bending); L0 (initial device luminance); L/L0 (the normalized luminance with varying bending cycles).

In order to test the mechanical flexibility of the prepared FOLED devices, the bending test was carried out, and a pre-purchased ITO-PET-based OLED was used for a comparison test, as shown in Figure 11. After bending 500 times, the peak luminance of the FOLED based on Ag NWs: Ag NPs/PEDOT: PSS/DMSO and the Ag NWs/PEDOT: PSS/DMSO anode still reached 82% and 70% of the initial luminance. However, the ITO-based devices were destroyed after 200 bending tests. This indicates that the FOLED prepared using Ag NWs: Ag NPs has obvious flexibility advantages.

## 4. Conclusions

In this study, PET/Ag NWs/PEDOT: PSS/DMSO FTEs were first prepared and optimized with three different methods, including methanol impregnation, argon plasma treatment, and ultraviolet radiation. To further improve the optoelectronic properties of the FTEs, a Ag NWs and Ag NPs co-doped system was adopted. The optimized PET/Ag NWs: Ag NPs/PEDOT: PSS/DMSO FTEs obtained an FOM value of 1.42 × 10^−2^ ohm^−1^ and a low sheet resistance of 13.86 ohm/sq. The FOLEDs based on the optimized FTEs exhibited a low turn-on voltage of 3.0 V, a luminous efficiency of 6.04 cd/A, and an EQE of 1.92%. Furthermore, it was noteworthy that there was no efficiency roll-off observed even when the maximum luminance was reached. At the same time, the prepared FOLEDs showed excellent mechanical flexibility, maintaining 82% of the initial luminance after 500 bending tests. These results indicate that FTE with the Ag NWs: Ag NPs co-doped system and the PEDOT: PSS composite structure is a favorable alternative to ITO for high-performance FOLED.

## Figures and Tables

**Table 1:** The average sheet resistance, transmittance at 550 nm, and $\sigma_{DC}/\sigma_{OP}\left(\lambda \right)$ value of FTEs processed by different methods.

Method	Average Sheet Resistance (ohm/sq)	Transmittance at 550 nm (%)	$\sigma_{\mathrm{DC}}/\sigma_{\mathrm{OP}}\left(\lambda \right)$
ITO	15	86	160.437
Methanol impregnation	14.72	83.855	139.1435
Argon plasma treatment	14.46	83.803	141.1259
Ultraviolet radiation	17.7	85.196	127.6885

**Table 2:** The specific performance values of the different FOLEDs prepared.

Device	Turn-On (V)	Luminance (cd/m^2^)	LE (cd/A) (Max)	EQE (%) (Max)
ITO	3.2	21830	4.10603	1.31403
Ag NWs: Ag NPs/PEDOT: PSS/DMSO	3.0	8622	6.03747	1.92450
Ag NWs/PEDOT: PSS/DMSO	3.1	8631	4.60186	1.55811

## Data Availability

Date are contained within the article.
